# Supplementary material for: Bifurcation in brain dynamics reveals a signature of conscious processing independent of report
Source: Nat Commun. 2021 Feb 19;12:1149. doi: 10.1038/s41467-021-21393-z (PMC7895979; doi:10.1038/s41467-021-21393-z)
Supplement: Supplementary file 1 — Supplementary Information [file 41467_2021_21393_MOESM1_ESM.pdf]

Supplementary Information

"Bifurcation in brain dynamics reveals a signature of conscious processing independent of report"

Authors: Claire Sergent[1,2,*], Martina Corazzol[1,2,¶], Ghislaine Labouret[1,2,3,¶], François Stockart[1,2], Mark Wexler[1,2], Jean-Rémi King[4], Florent Meyniel[5], Daniel Pressnitzer[4]

Affiliations:

[1]Université de Paris, 75006 Paris, France.

[2]CNRS (Integrative Neuroscience and Cognition Center, UMR 8002), Paris, France.

[3]Laboratoire de Sciences Cognitives et Psycholinguistique, Département d'Etudes Cognitives, École normale supérieure, EHESS, CNRS, PSL University, Paris, France

[4]Laboratoire des Systèmes Perceptifs, Département d'études cognitives, École normale supérieure, PSL University, CNRS, 75005 Paris, France.

[5]Cognitive Neuroimaging Unit, CEA, INSERM, Université Paris-Sud, Université Paris-Saclay, NeuroSpin center, 91191 Gif/Yvette, France

*Correspondence to: claire.sergent@u-paris.fr

¶ These authors contributed equally

Content: 7 supplementary figures

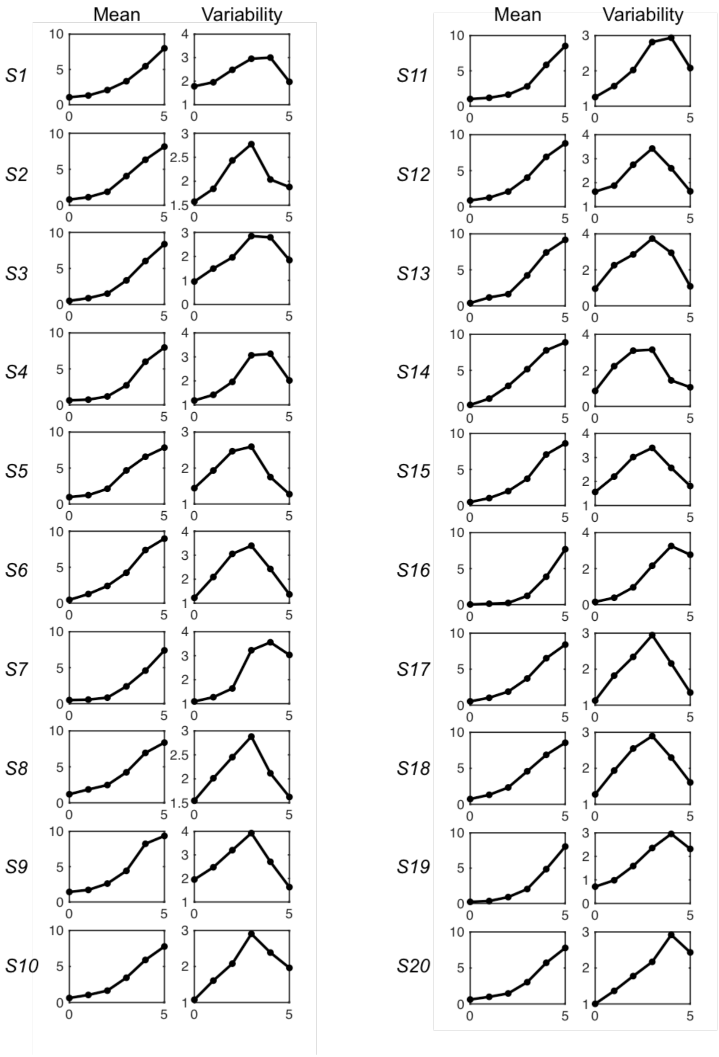

Supplementary Figure 1. Behavioral profiles: mean and variability of subjective audibility for each participant. For each participant we represent the mean audibility across trials (left graph) and the variability of audibility across trials (standard deviation, right graph) for the 6 stimulation levels (0= noise, 5= highest SNR of -5dB). Source data for this figure are provided as a Source Data file.

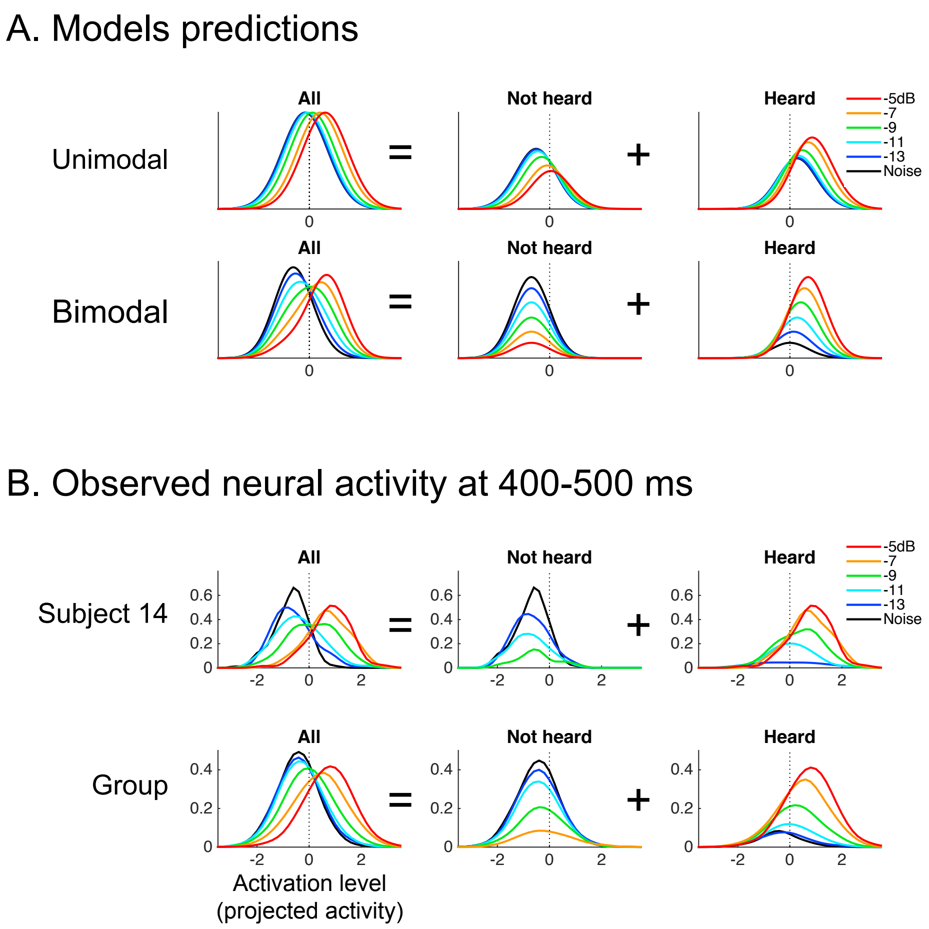

Supplementary Figure 2. Predicted and observed distributions of activity across trials in the active condition. Predicted distributions of activity as a function of SNR level and behavioral report for unimodal non-linear and bifurcation dynamics (A) can be compared to distributions of neural activity observed within the 400-500ms time window for subject 14 or at the group level (B). Neural activity (projected activity) is z-scored for each subject before calculating the different distributions. Distributions for each participant are derived using kernel density estimation. These distributions are then averaged across participants to obtain distributions for the group. Source data for this figure are provided as a Source Data file.

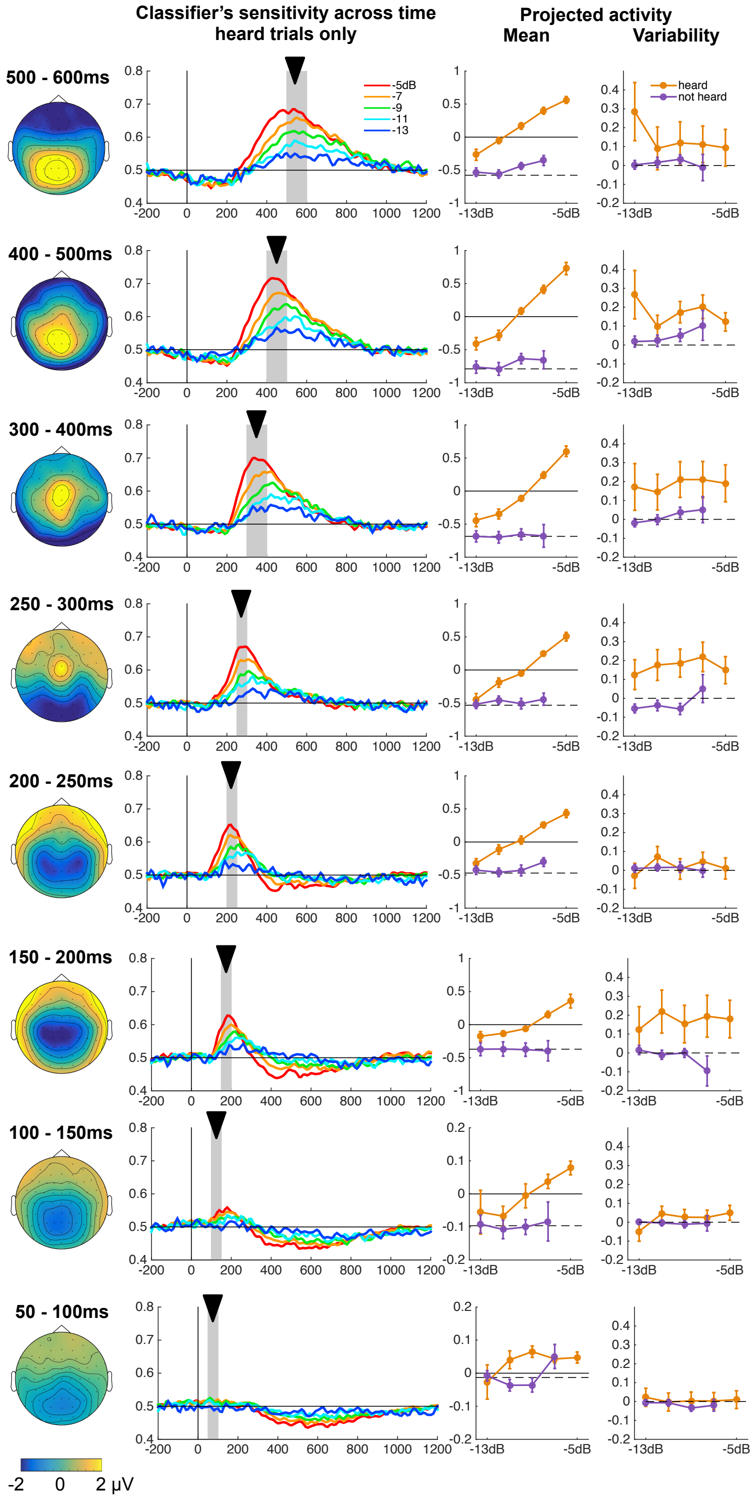

Supplementary Figure 3. Dynamics of the different processing stages during the active session, separately for heard and not-heard trials. The left column represents the group average topographies at each time window for the strongest SNR (-5dB; same as in Fig.4.), the next column shows the group average time courses of these processes for the different SNRs for heard trials only: note that, compared to Fig2., the amplitude of these different stages processes appear to increase linearly with SNR. The third column shows the group average profile of mean classification score (AUC) with SNR for each time window of interest, separately for heard and not heard trials, confirming that the profile is roughly linear

for heard trials. The last column shows the group average profile of variability with SNR separately for heard and not-heard trials. The non-monotonic profile observed in Fig.2. is abolished, confirming that this profile and the underlying burst of variance at consciousness threshold is due to a split in the distribution of activity between conscious and unconscious trials. Error bars correspond to ± SEM, n = 20 participants. Source data for this figure are provided as a Source Data file.

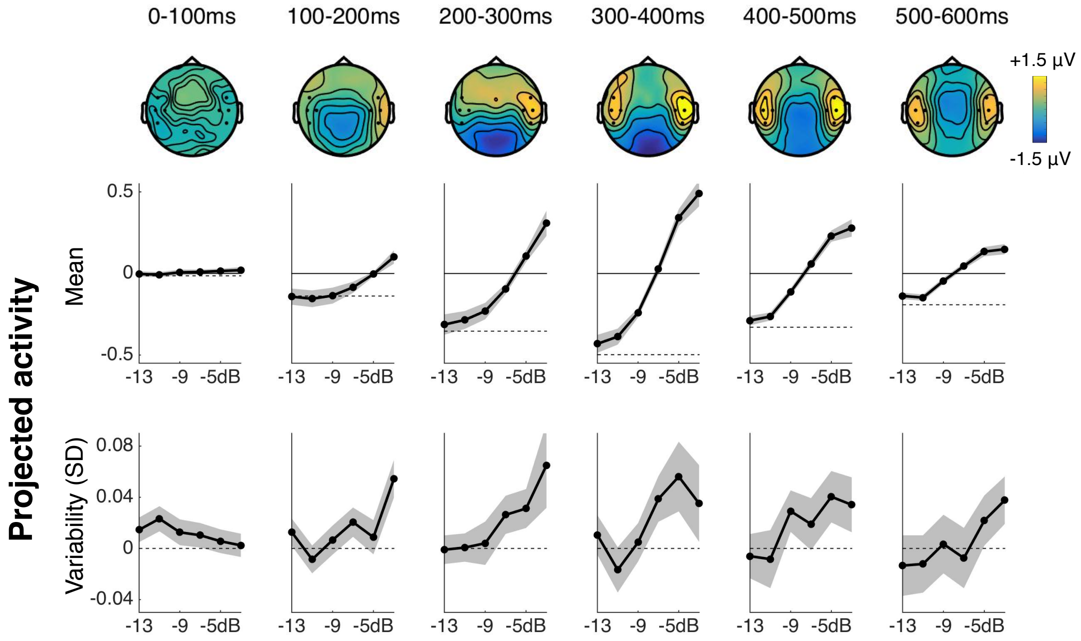

Supplementary Figure 4. Neural dynamics for passive sessions. The top row shows the group averaged topographies at different time windows following targets played at -5 dB SNR. The middle and bottom rows show the mean and variability of projected activity as a function of SNR, for the different time windows and over a group of temporal electrodes highlighted on the topographies. Shaded areas are SEMs. Source data for this figure are provided as a Source Data file.

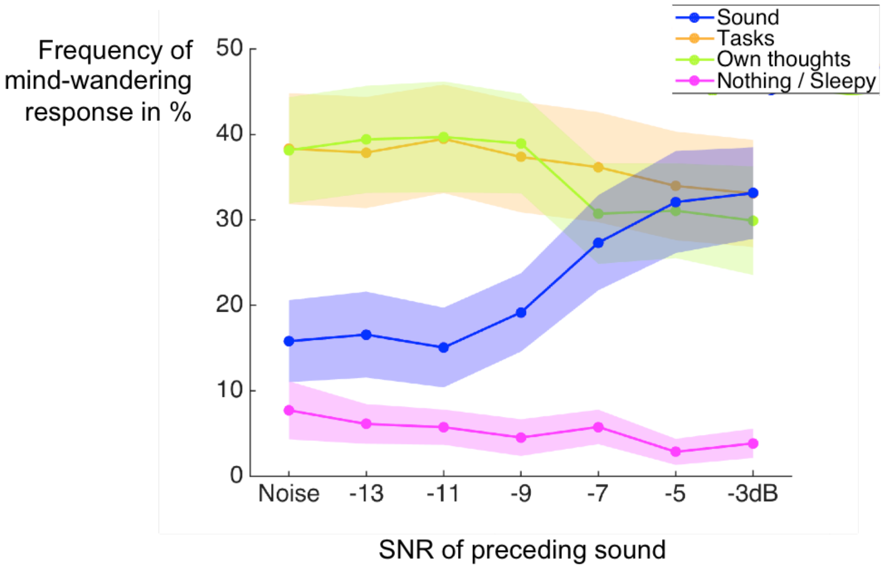

Supplementary Figure 5. Responses to mind-wandering probes during the passive sessions: frequencies of the four response categories as a function of the SNR of the preceding sound. The points show the average across participants, the shaded areas represent ±SEM. Source data for this figure are provided as a Source Data file.

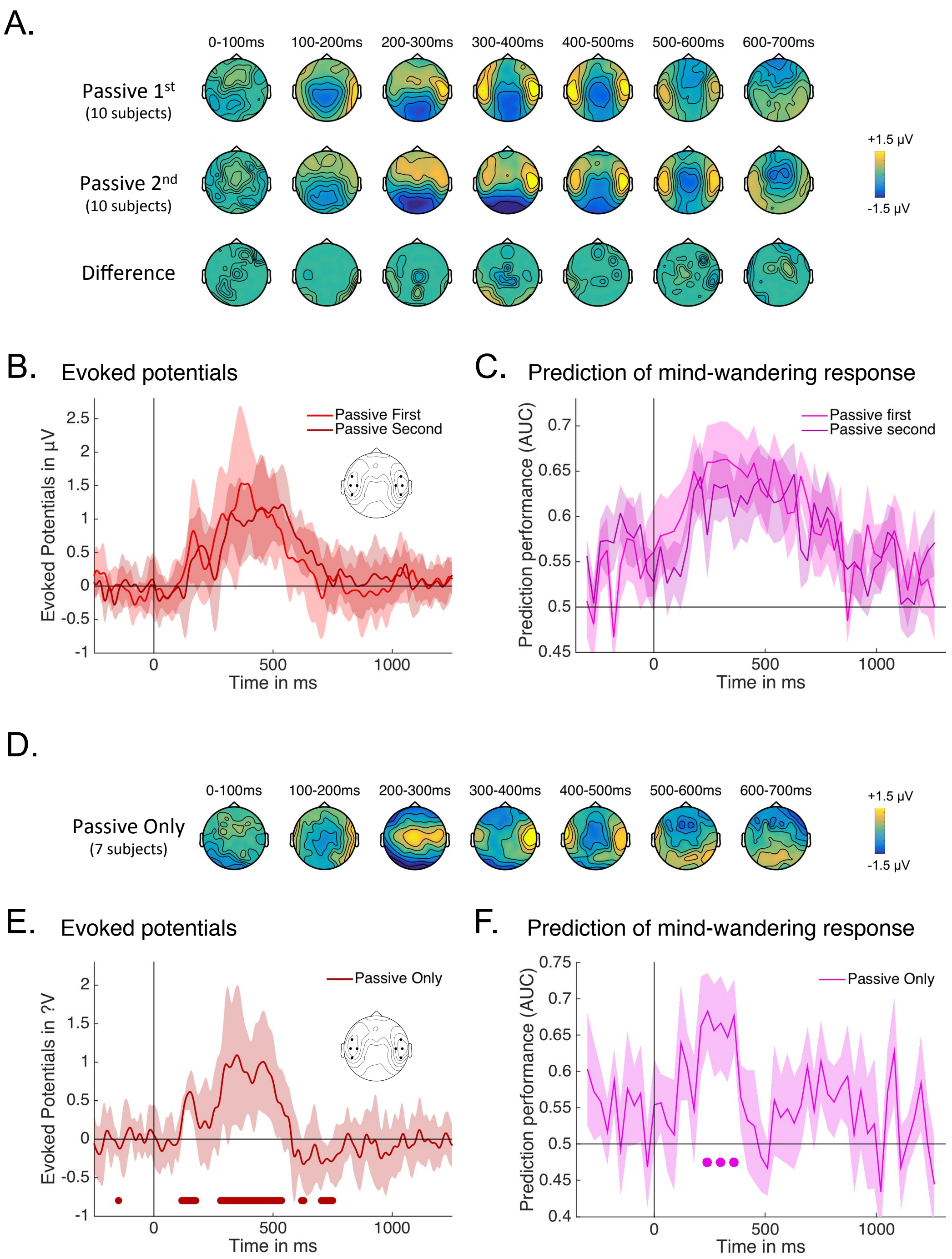

Supplementary Figure 6. Probing potential effects of the active session on brain activity in the passive session

(A) Group averaged brain topographies recorded in the passive sessions for subjects who undertook this session first (10 subjects) or second (10 subjects). The bottom topographies indicatively show the electrodes where a difference was observed between the two groups at p-uncorrected < 0.05; none of these effects survived correction for multiple comparison. (B) Time course of the evoked potentials over temporal electrodes (shown on the glass topography). Shaded areas represent SEMs (n = 10 independent participants for each condition). No significant difference was observed between the two groups. (C) Time courses

of the prediction of mind-wandering responses (sound versus other). Shaded areas represent SEMs (n = 10 independent participants for each condition). No significant difference was observed between the two groups. (D-F) Same graphs for the participants of control experiment 1, who only undertook a passive session. Shaded areas represent SEMs (n = 7 parcitipants). Source data for this figure are provided as a Source Data file.

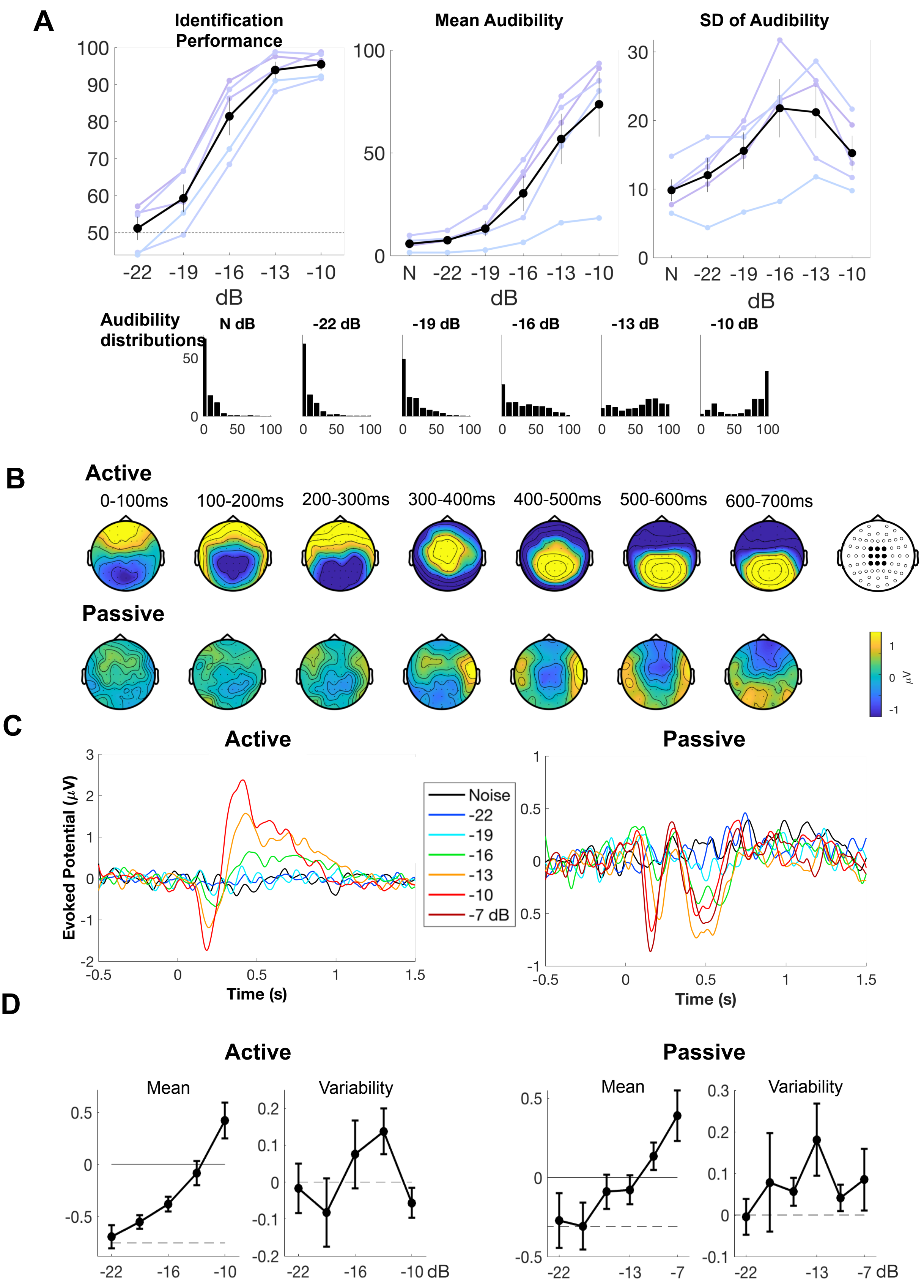

Supplementary Figure 7. Results of control experiment 2, with pure tones (5 participants who undertook both an active and a passive session). (A) Behavioral results for the "Tones" active session: identification performance (high vs low tones), mean audibility, standard deviation of audibility across trials and the audibility distributions are

plotted as a function of SNR. The behavioral results are presented for each participant with a faint colored line and averaged across participants with a thick black line. Error bars represent ± SEM, n = 5 participants. (B) Topographies for the active (top) and passive (bottom) sessions, The group average topographies are presented at different time windows for tones played at the highest SNR (-10dB for active and -7dB for passive). (C) Group average evoked potentials for the active and passive sessions, with color-coded SNRs. Evoked potentials were computed over a central region of interest (ROI, highlighted by black dots on panel B). (D) Mean and variability (inter-quartile range) of projected activity across trials as a function of SNR (variability on the "noise" trials served as a baseline) in the active and passive sessions in the time window 250-300ms. Error bars correspond to ± SEMs, n = 5 participants. Source data for this figure are provided as a Source Data file.
