# Supplementary material for: Bifurcation in brain dynamics reveals a signature of conscious processing independent of report
Source: Nat Commun. 2021 Feb 19;12:1149. doi: 10.1038/s41467-021-21393-z (PMC7895979; doi:10.1038/s41467-021-21393-z)
Supplement: Supplementary file 3 — Reporting Summary [file 41467_2021_21393_MOESM3_ESM.pdf]

nature research

Corresponding author(s): Claire Sergent

Last updated by author(s): Oct 30, 2020

Reporting Summary

Nature Research wishes to improve the reproducibility of the work that we publish. This form provides structure for consistency and transparency in reporting. For further information on Nature Research policies, see our Editorial Policies and the Editorial Policy Checklist.

Statistics

For all statistical analyses, confirm that the following items are present in the figure legend, table legend, main text, or Methods section.

n/a	Confirmed	
☐	☒	The exact sample size (*n*) for each experimental group/condition, given as a discrete number and unit of measurement
☐	☒	A statement on whether measurements were taken from distinct samples or whether the same sample was measured repeatedly
☐	☒	The statistical test(s) used AND whether they are one- or two-sided<br>*Only common tests should be described solely by name; describe more complex techniques in the Methods section.*
☐	☒	A description of all covariates tested
☐	☒	A description of any assumptions or corrections, such as tests of normality and adjustment for multiple comparisons
☐	☒	A full description of the statistical parameters including central tendency (e.g. means) or other basic estimates (e.g. regression coefficient) AND variation (e.g. standard deviation) or associated estimates of uncertainty (e.g. confidence intervals)
☐	☒	For null hypothesis testing, the test statistic (e.g. *F*, *t*, *r*) with confidence intervals, effect sizes, degrees of freedom and *P* value noted<br>*Give P values as exact values whenever suitable.*
☐	☒	For Bayesian analysis, information on the choice of priors and Markov chain Monte Carlo settings
☒	☐	For hierarchical and complex designs, identification of the appropriate level for tests and full reporting of outcomes
☐	☒	Estimates of effect sizes (e.g. Cohen's *d*, Pearson's *r*), indicating how they were calculated

Our web collection on statistics for biologists contains articles on many of the points above.

Software and code

Policy information about availability of computer code

Data collection	The vowel stimuli were synthetized using MBROLA v3.02b. The stimuli were presented using the Matlab-based Psychtoolbox (http://psychtoolbox.org/) running under Matlab 2014b. EEG recordings were carried out using the Brain Products' Brain Vision Recorder v 2016 for the main experiment and v 2019 for control experiments.
Data analysis	All the toolboxes used to analyze the data are described in the methods section of the manuscript: data analysis was carried out using the Matlab based fieldtrip toolbox (http://www.fieldtriptoolbox.org/, v 2018), the Matlab based Brainstorm toolbox (http://neuroimage.usc.edu/brainstorm, v 2018-2019), the Python based MNE-Python toolbox (https://martinos.org/mne/stable/index.html, v 2018), the Matlab based EEGlab toolbox, including FASTER toolbox (EEGLAB/eeglab14_1_2b, FASTER v1.2.3b), as well as custom made scripts in Matlab (v. R2014_B and v R2018b for preprocessing of the control experiments) and Python (3.5)

For manuscripts utilizing custom algorithms or software that are central to the research but not yet described in published literature, software must be made available to editors and reviewers. We strongly encourage code deposition in a community repository (e.g. GitHub). See the Nature Research guidelines for submitting code & software for further information.

Data

Policy information about availability of data

All manuscripts must include a data availability statement. This statement should provide the following information, where applicable:

- Accession codes, unique identifiers, or web links for publicly available datasets
- A list of figures that have associated raw data
- A description of any restrictions on data availability

Data availability statement: at the moment the datasets generated during and/or analyzed during the current study are available from the corresponding author on reasonable request. [These data will be deposited into a public repository and the accession codes will be available before publication]

Field-specific reporting

Please select the one below that is the best fit for your research. If you are not sure, read the appropriate sections before making your selection.

- [x] Life sciences
- [ ] Behavioural & social sciences
- [ ] Ecological, evolutionary & environmental sciences

For a reference copy of the document with all sections, see nature.com/documents/nr-reporting-summary-flat.pdf

Life sciences study design

All studies must disclose on these points even when the disclosure is negative.

Sample size	Following recommendations for EEG studies (S. J. Luck, An introduction to the event-related potential technique. The MIT Press, Cambridge, Massachusetts, ed. Second edition., 2014.) and previous studies (eg A. Del Cul, S. Baillet, S. Dehaene, Brain dynamics underlying the nonlinear threshold for access to consciousness. PLoS Biol 5, e260 (2007).), the total number of participants required was estimated at 20.
Data exclusions	Twenty-five native French speakers aged 18 to 30 years took part in the experiment. Two chose to discontinue participation before the end, and three were excluded, following pre-established criteria, on the basis of too many artifacts in the EEG recordings (more than 25% of trials containing an artifact). The remaining 20 participants, all right-handed, included 10 women, and had a mean age of 23.4 years (range 21-29).
Replication	The present study includes one replication of the passive session of the main experiment (10 new participants). The results were successfully replicated (Fig. S6). The stydy also includes a replication using different stimuli (tones instead of vowels) for both passive and active sessions (5 new participants that took both sessions). The results were successfully replicated (Fig. S7).
Randomization	The main results of this study involve within group comparisons. The only between group comparison concerns the order of active and passive session; the attribution to each group was random
Blinding	All the participants took all the conditions. Within each active or passive session,the experimental conditions were randomized across trials by the computer, both subjects and expreimenters were thus blind to the conditions that were presented at each trial. The order of the sessions was counterbalanced accross participants. Both participant and subject knew whether they were performing the passive or the active session because the condition itself requires to inform the participant as to whether they should perform a task on the stimulus or not: it is the effect of these instructions that is tested when comparing these conditions.

Reporting for specific materials, systems and methods

We require information from authors about some types of materials, experimental systems and methods used in many studies. Here, indicate whether each material, system or method listed is relevant to your study. If you are not sure if a list item applies to your research, read the appropriate section before selecting a response.

Materials & experimental systems

n/a	Involved in the study	
[x]	[ ]	Antibodies
[x]	[ ]	Eukaryotic cell lines
[x]	[ ]	Palaeontology and archaeology
[x]	[ ]	Animals and other organisms
[ ]	[x]	Human research participants
[x]	[ ]	Clinical data
[x]	[ ]	Dual use research of concern

Methods

n/a	Involved in the study	
[x]	[ ]	ChIP-seq
[x]	[ ]	Flow cytometry
[x]	[ ]	MRI-based neuroimaging

Human research participants

Policy information about studies involving human research participants

Population characteristics	Twenty-five native French speakers aged 18 to 30 years took part in the experiment. Two chose to discontinue participation before the end, and three were excluded on the basis of too many artifacts in the EEG recordings (more than 25% of trials containing an artifact). The remaining 20 participants, all right-handed, included 10 women, and had a mean age of 23.4 years (range 21-29).
Recruitment	Participants were recruted via a French Database for volunteers in Cognitive Research. Particpants are tmostly students in higher education. Our study bearing on very general aspects of auditory perception, this should not have biased the results in any way.
Ethics oversight	The study was validated by the ethics committee of Paris Descartes (CERES).

Note that full information on the approval of the study protocol must also be provided in the manuscript.
